# Implications of Gender on the Outcome in Patients With Autoimmune Hepatitis

**DOI:** 10.7759/cureus.55477

**Published:** 2024-03-04

**Authors:** Sayan Malakar, Samir Mohindra, Piyush Mishra, Srikanth Kothalkar, Vivek V Shirol, Gourav Borah, Umair Shamsul Hoda, Nishant Shah, Kartik Balankhe, Gaurav Pande, Uday C Ghoshal

**Affiliations:** 1 Department of Gastroenterology, Sanjay Gandhi Postgraduate Institute of Medical Sciences, Lucknow, IND

**Keywords:** autoimmune flare up, seronegative autoimmune hepatitis, chronic liver disease (cld), live cirrhosis, acute on chronic liver disease, aih -autoimmune hepatitis

## Abstract

Background: Autoimmune hepatitis (AIH) is uncommon and predominantly affects females. Data on AIH from India are scanty. We retrospectively analyzed the spectrum and outcome of adults with AIH and compared it between male and female patients.

Methods: AIH was diagnosed using a simplified AIH score. For suspected seronegative AIH, the revised score was used. Standard therapies for AIH and portal hypertension were administered and response was assessed at six months. Relapse rates and five-year mortality were also evaluated.

Results: Of the 157 patients with AIH, 85 (male: female 25: 60) were included in the study. The median age at diagnosis was 46 (interquartile range (IQR) 32-55.5) years in males vs 45 (IQR 34.2-54) years in females (p=0.91). A similar proportion of male and female patients presented with cirrhosis, acute severe AIH, or AIH-related acute on chronic liver failure (ACLF); Extra-hepatic autoimmune diseases were less common in male patients (16% vs 35.5% p=0.02). Other laboratory and histological features were comparable in both groups. During the median follow-up period of 51 months (IQR 45-67 months). The biochemical and clinical response at six months were seen in 64% of male patients and 63.3% of female patients (p= 0.57). Of patients, 75% relapsed in the male AIH group (12 of 16 patients) after initial remission compared to 42% in the female group (p=0.02). Five-year mortality was 14.1%, and no patient developed hepatocellular carcinoma.

Conclusion: Male and female patients with AIH have similar clinical, biochemical, and histological profiles. More male patients relapsed after an initial response to therapy.

## Introduction

Autoimmune hepatitis (AIH) is an uncommon cause of chronic liver disease (CLD) [1]. AIH predominantly affects females; however, the incidence of AIH is also increasing among males in Asian countries [2]. There are other significant differences in demographic, clinical, biochemical profile, and treatment response in Asian patients compared to the Western population [3]. While most patients with AIH present with long-standing fatigue and jaundice, a significant number of patients are asymptomatic [4]. Acute presentation in AIH is seen in 15-25% of patients and cirrhosis is present in 30% of patients with AIH [5]. Previous studies have shown that there might be significant differences in demographic, clinical, laboratory profile, and outcomes between male and female patients with AIH [3,6]. However, data on AIH is limited from India. So in this study, we aimed to characterize the demographic, clinical, biochemical, and histological profile of patients with AIH. We have also compared the outcome among male and female patients with AIH.

## Materials and methods

This was a single-center retrospective study conducted at Sanjay Gandhi Post Graduate Institute of Medical Sciences, a university hospital located in northern India. The study was approved by the Sanjay Gandhi Postgraduate Institute of Medical Sciences Institutional Ethics Committee (approval number: IEC-2024-18-DM-EXP-56). Patients with autoimmune hepatitis, evaluated between 2015 and 2020, were included in our study. All patients underwent thorough evaluation for their underlying liver disease. Patients with suspected alternative etiologies were not included in the study even if they had some biopsy evidence of AIH (like interface hepatitis, emperipolesis, or lymphoplasmacytic infiltration).

AIH was diagnosed using a simplified AIH score; for suspected seronegative AIH, a revised score was used [7]. All biopsy-proven AIH patients who had simplified AIH scores ≥ 6 were included in the study. AIH-related acute liver failure (ALF), acute-on-chronic liver failure (ACLF), and acute severe AIH (AS-AIH) were diagnosed using standard diagnostic criteria [8].

Patients who were excluded from the analysis (despite fulfilling a simplified AIH score more or equal to 6) include those with alcohol intake > 50 grams/day, chronic hepatitis B and C, underlying metabolic syndrome and significant steatosis on liver biopsy, suspected drug-induced liver injury, and drug-induced AIH-like liver injury.

Patients with cirrhosis, AS-AIH, and ACLF were treated with prednisolone. Non-cirrhotic AIH patients also received budesonide [8]. All patients received standard treatment for liver disease-related complications. The median follow-up period was 51 months (interquartile range (IQR) 45-67).

The treatment response was defined after six months according to the consensus by the International Autoimmune Hepatitis Group (IAIHG) collaborators’ consensus [9]. The biochemical response was defined by the reduction of aspartate aminotransferase (AST), and alanine aminotransferase (ALT) at six months. The normalization of liver enzymes and immunoglobulin G (IgG) was considered the complete biochemical response [9]. Reduction of IgG and liver enzymes from the baseline but not below the complete response range was counted as an incomplete response [9]. Relapse was defined as the recurrence of symptoms after the cessation of therapy associated with elevated liver enzymes. Potential alternative etiologies during relapse were ruled out [9]. Patients who did not respond to or developed drug-related complications to the first line therapy second-line were considered [9].

Statistical analysis

Data were maintained in an Excel sheet (Microsoft Corporation, Redmond, Washington, United States). Data were analyzed in IBM SPSS Statistics for Windows, Version 20.0 (Released 2011; IBM Corp., Armonk, New York, United States). The data of male and female patients were compared using standard statistical tests. Continuous variables were calculated as median (IQR) and categorical variables as numbers (percentages). Chi-square or Fisher’s exact tests were used to compare categorical parameters and Student’s t-test for continuous parameters. Overall survival and relapse-free survival were estimated using Kaplan-Meier analysis. 

## Results

Patient demography and clinical presentation

**Figure 1:**
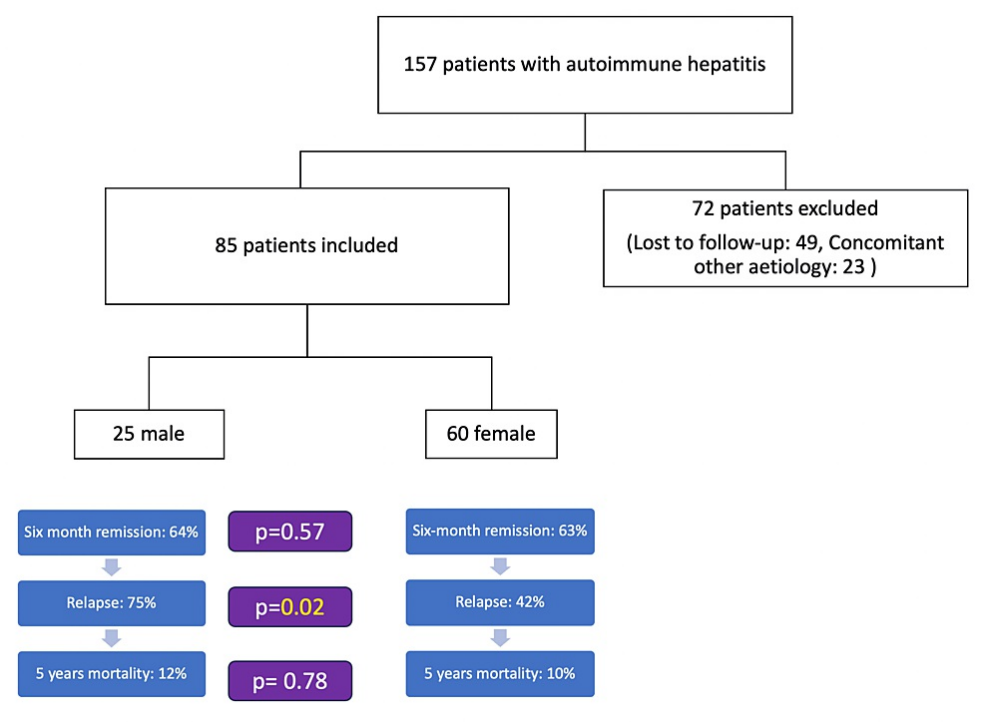
Flow chart showing the inclusion and the exclusion criteria with the outcome

A total of 157 patients were diagnosed with AIH. Of these, 72 (45%) were excluded and finally, a total of 85 patients were included in the analysis (Figure 1). The majority of the patients were females (male:female = 25:60). The median age at diagnosis was 46 (IQR 32-55.5) years in males vs 45 (IQR 34.2-54) years in females. It was not statistically significant. More male patients were asymptomatic and were found to have AIH while working up for other issues (four patients (16%) in males vs. three (5%) in female patients; p = 0.10). Among the symptomatic patients, jaundice was the most common presenting complaint present in 71 patients (83.5%). Portal hypertension (PHTN)-related bleeding, hepatic encephalopathy (HE), and ascites were present in four (4.7%), 10 ( 11.7%), and seven (12.1%) patients, respectively. There were no significant differences in these presentations between male and female patients (Table 1). Seven (8%) patients had a history of pruritus but they did not have any evidence of overlap on biopsy. On presentation, 23 (27%) patients had cirrhosis (five (20%) in males vs 18 (30%) in female patients; p= 0.25). A comparable number of patients also presented with ACLF (four males (16%) vs six (10%) females; p=0.33) and AS-AIH and ALF; there are no significant differences among them. Autoimmune diseases were present in four (16%) male patients which was lower compared to females (16% vs 35%; p=0.02).

**Table 1:** Demographic and clinical profile of patients with autoimmune hepatitis ACLF: acute-on-chronic liver failure; AS-AIH: acute severe autoimmune hepatitis; ALF: acute liver failure

Clinical	Male (n=25), n (%)	Female (n=60), n (%)	p-value	Chi-square value
Age	46 (32-55.5)	45 (34.2-54)	0.91	35.6
Asymptomatic	4 (16%)	3 (5%)	0.10	2.80
Jaundice	19 (78%)	52 (86%)	0.18	1.45
GI bleed	2 (8%)	2 (3.3%)	0.33	0.85
Hepatic encephalopathy	4 (16%)	6 (10%)	0.33	0.61
Ascites	2 (8%)	5 (8.3%)	0.66	0.03
Pruritus	1 (4%)	6 (10%)	0.33	0.84
Varices	14 (58.3%)	27 (45%)	0.19	0.81
Other autoimmune diseases	4 (16%)	21 (35.6%)	0.02	1.22
Cirrhosis	5 ( 20%)	18 (30%)	0.25	1.63
Chronic Hepatitis	9 (36%)	39 ( 45%)	0.18	0.89
AS-AIH	6 (24%)	17 (28.3%)	0.45	0.16
ALF	1 (4%)	5 (8.3%)	0.47	0.76
ACLF	4 (16%)	6 (10%)	0.33	0.61

Laboratory parameters

Routine biochemical parameters did not differ significantly between the two groups. The mean bilirubin was 2.1 (1.1-17.1) mg/dL for males and 2.8 (1.3-6.8) mg/dL for female patients (p value=0.66), AST was higher than ALT in both the groups, but there were no differences between the male and female groups. Serum AST, ALT, ALP, albumin, and IgG were lower in the male patients. Still, there were no significant differences (Table 2). Antinuclear antibody (ANA) was the most prevalent (76%) autoantibody (88% in males vs 71.7% in females; p value=0.08), and anti-smooth muscle antibody (ASMA) was the second most common (55%) antibody found in AIH (Table 2). Anti-neutrophilic cytoplasmic antibody (ANCA) and anti-mitochondrial (AMA) were positive in 7% and 8% of patients with AIH, respectively. Still, there was no evidence of any overlap syndromes among them. 

**Table 2:** Laboratory parameters ALP: alkaline phosphatase; ALT: alanine aminotransferase; AST: aspartate aminotransferase; IgG: immunoglobin G

Patient characteristics	Male (n=25), Median (IQR)	Female (n=60), Median (IQR)	p-value	t-value
Hemoglobin (g/dL)	9.8 (9.2-12.1)	10.9 (9.6-11.9)	0.20	1.11
Total leukocyte count (per/mm^3^)	6000 (2700-10000)	4850 (3850-7730)	0.49	-0.68
Platelet count (lacs/mm^3^)	1.1 (0.6-1.42)	1.27 (0.81-1.6)	0.51	1.04
Total Bilirubin mg/ dL	2.1 (1.1-17.1)	2.8 (1.3-6.8)	0.82	-0.32
Conjugated Bilirubin	0.7 (0.5-10)	1.25 (0.62-4)	0.62	-0.48
AST (U/L)	121 (59-303)	121 (64-304)	0.66	0.51
ALT (U/L)	94 (37-139)	79 (47-198)	0.33	0.29
ALP (U/L)	163 (107-183)	176 (109-284)	0.21	1.49
Albumin (g/dL)	3.3 (2.9-4.0)	3.4 (2.8-3.8)	0.63	-0.47
Protein	7.8 (7-8.1)	7.3 (6.4-8.1)	0.93	0.76
IgG	2662 (1450-2860)	2858 (1540-2630)	0.51	0.85
INR	1.49 (1.2-1.6)	1.3 (1.1-1.8)	0.65	-0.48

Seronegative AIH

Overall, six patients (7%) were diagnosed with seronegative AIH (all female) (Table 3). Six patients underwent anti-soluble liver antigen (anti-SLA), all negative. And anti-liver cytosol-1 (LC) was done in four which also came to be negative (Table 3). Among seronegative AIH patients, two presented with AS-AIH, three with ACLF, and one with chronic hepatitis. Four of them responded to immunosuppressive therapy and two died of liver-related decompensation.

**Table 3:** Antibody profile in patients with autoimmune hepatitis AMA: anti-mitochondrial antibody; ANA: anti-nuclear antibody; ANCA: antineutrophil cytoplasmic antibody; ASMA: anti-smooth muscle antibody

Antibody	Male (n=25), n (%)	Female (n=60), n (%)	p-value	Chi-square value
ANA	22 (88%)	43 (71.7%)	0.08	18.7
ASMA	15 (60%)	32 (68%)	0.37	17.5
ANCA	2 (8%)	4 (6.7%)	0.82	0.31
AMA	0	7 (11.7%)	0.07	0.25
Seronegative AIH	0	6 (10%)	0.10	2.56

Histological parameters

Moderate interface hepatitis (IH) was the most common finding on biopsy, present in 79 (93%) patients (96% in male patients and 91.7% in female patients; p=0.47). Apart from that, lymphoplasmacytic infiltration, emperipolesis, necrosis, and rosette formation were present in 33 (50.5%), 23 (27%), 19 (22%), and 11 (13%) patients in AIH, respectively. The differences weren’t statistically significant when comparing male and female patients (Table 4).

**Table 4:** Histological profile

Histology	Male (n=25), n (%)	Female (n=60), n (%)	p-value	Chi-square value
Moderate Interface hepatitis	24 (96%)	55 (91.7%)	0.47	0.32
Lymphoplasmacytic infiltration	11 (44%)	32 (53%)	0.62	2.50
Emperipolesis	5 (20%)	18 (30%)	0.34	0.43
Rosette	4 (16%)	7 (11.7%)	0.58	0.29
Necrosis	6 (24%)	13 (21.7%)	0.78	0.10

Response to treatment and relapse

**Figure 2:**
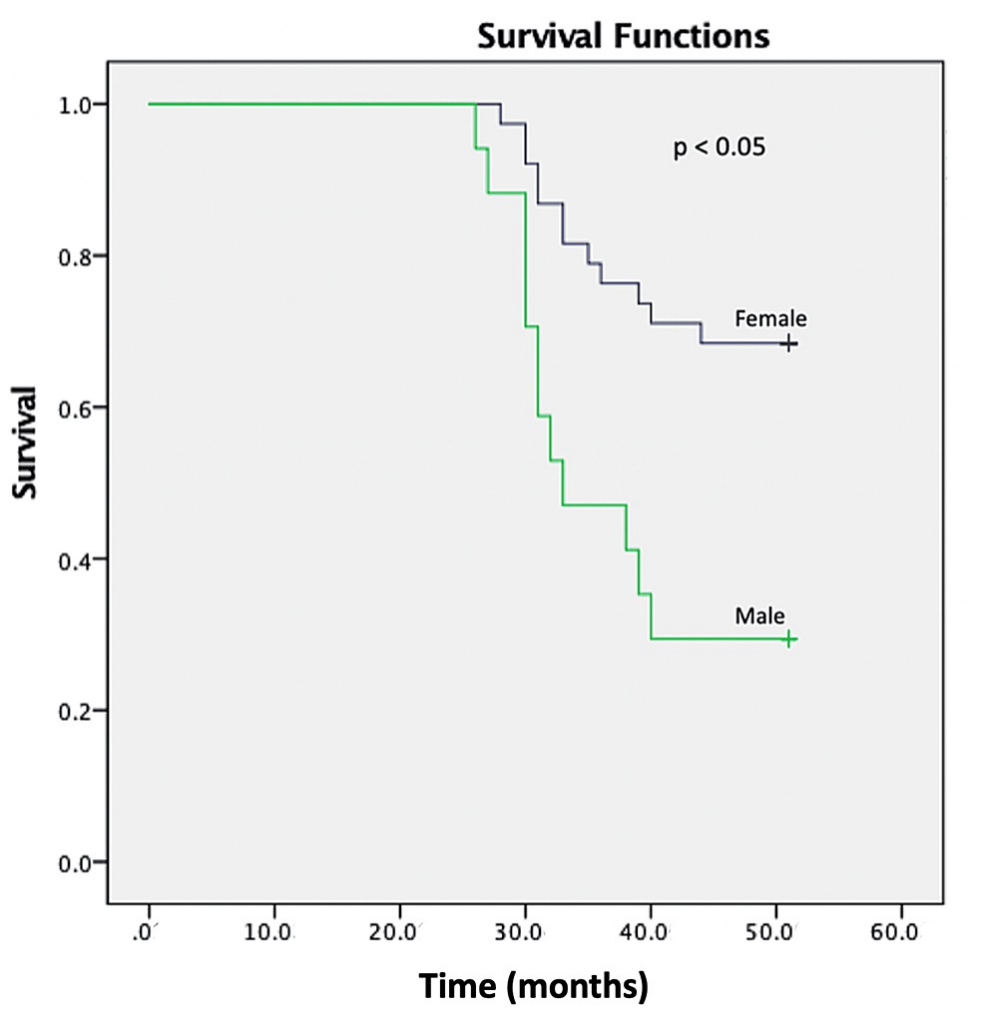
Relapse-free survival in male and female patients with autoimmune hepatitis by log-rank test (p= 0.002)

A total of 23 (92%) male patients with AIH received immunosuppressive therapy (16 (64%) patients received steroid plus azathioprine and seven (28%) received steroid alone) along with standard treatment for portal hypertension. Biochemical response at six months was seen in 54 (63.5%) patients (15 male patients (63.3%) vs 38 (64%) female patients; p=0.57). The complete biochemical response was seen in 10 (40%) male patients and 27 (45%) female patients (p= > 0.05). Among 16 male patients with AIH who initially responded, 12 of them (75%) had a relapse compared to 11 (42%) in female patients with AIH. It was statistically significant (p=0.02) (Table 5, Figure 2). We did not find any other parameters to predict relapse in patients with AIH.

**Table 5:** Treatment outcome of patients with autoimmune hepatitis Data given as n (%) and median (IQR) as indicated

Treatment outcome	Male (n=25)	Female (n=60)	p-value	Chi-square value
Biochemical and clinical response rate at six months, n (%)	16 (64%)	38 (63.3%)	0.57	20.6
Relapse, n (%)	12/16 (75%)	16/38 (42%)	0.02	12.8
Time to relapse after stopping or de-escalating the drugs (months), median (IQR)	9.5 (4-13.5)	8.6 (6-11.75)	0.14	0.78
Five-year mortality, n (%)	4 (16%) )	8 (13.3%)	0.78	0.03

**Figure 3:**
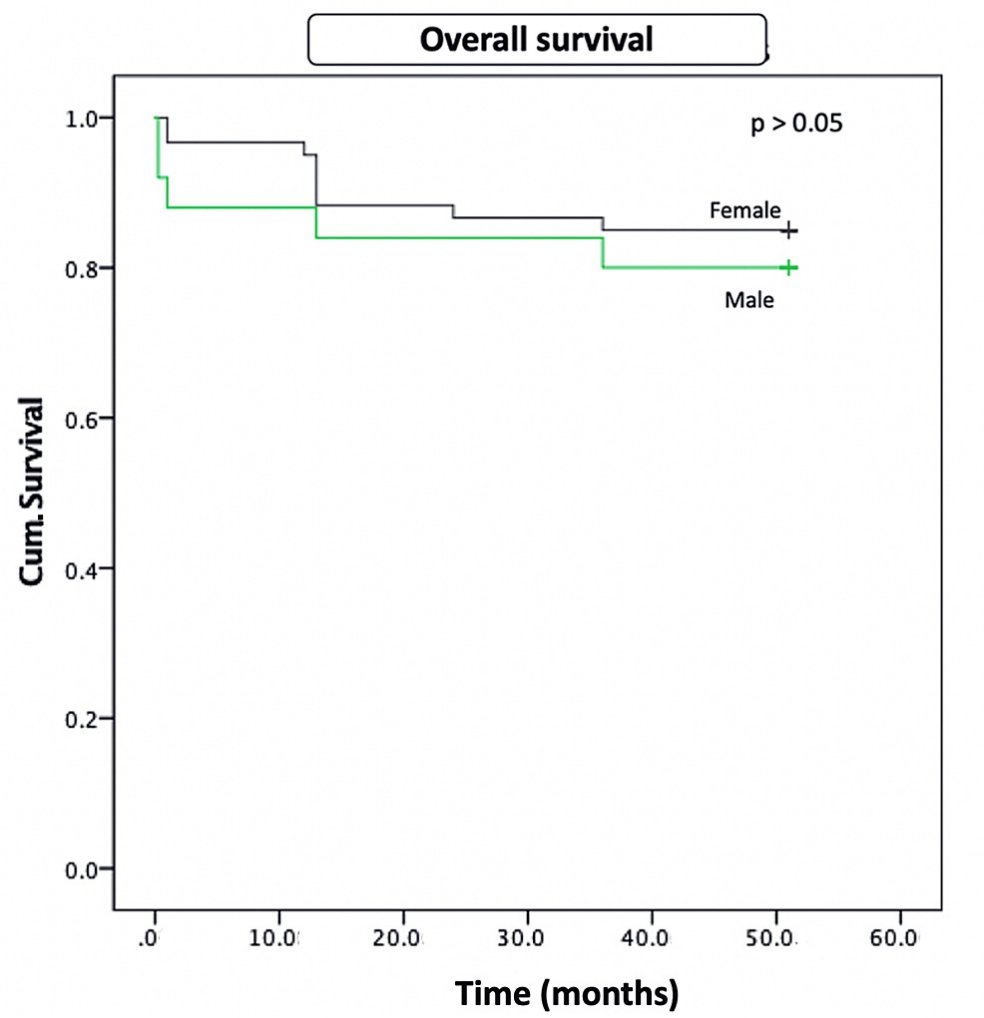
Cumulative survival in male and female patients with autoimmune hepatitis by log-rank test (p= 0.52)

The median time of relapse was six months (IQR: 4-10.5 months), There was no difference in time to relapse for both groups (p=0.14). Five-year mortality in male patients with AIH is 16% compared to 13% in their female counterparts. It was not statistically significant (Figure 3). 

Acute severe autoimmune hepatitis

Six male (24%) and 17 (28%) female patients presented with AS-AIH (n=23, 27%). Median age of presentation was 43 years (IQR: 35-56). The median age of the presentation and laboratory parameters were similar. Remission was seen in three (50%) male and 11 (64%) female patients, respectively (p=0.71). Overall mortality was 26%. Two female patients required liver transplantation. Two of three male patients (67%) and nine of 13 female (69%) female patients had a relapse after initial therapy (Table 6).

**Table 6:** Baseline features of patients with acute-severe autoimmune hepatitis ALP: alkaline phosphatase; ALT: alanine aminotransferase; AST: aspartate aminotransferase; IgG: immunoglobin G; INR: international normalized ratio

Patient characteristics	Male (n=6), Median (IQR)	female (n=17), Median (IQR)
Age	50 (25-57)	49 (36-58)
Hemoglobin (g/dL)	9.55 (8.0-10.9)	10.7 (9.25-11.97)
Total leukocyte count (per/mm^3^)	5450 (1951-10750)	4755 (9250-7950)
Platelet count (lacs/mm^3^)	1.11 (0.9-1.29)	1.52 (1.27-1.74)
Total bilirubin mg/ dL	18 (1.85-22.2)	3.41 (1.35-6.9)
Conjugated bilirubin	13 (3.9-17)	1.55 (0.57-4)
AST (U/L)	121 (142-458)	146 (62.75-305)
ALT (U/L)	62 (134-234)	79 (45-317)
ALP (U/L)	136 (78-335)	202 (108-383)
Albumin (g/dL)	2.9 (2.45-3.55)	3.6 (2.7-3.6)
Protein	7.8 (6.7-8.12)	7.15 (6.4-8.1)
IgG	2050 (2080-3546)	2655 (2010-3545)
INR	1.37 ( 1.11-1.82)	1.44 (1.06-1.70)
Outcome		
Remission (complete and incomplete)	50% (3/6)	64% (11/17)
Mortality	33% (2/6)	23% (4/17)
Relapse	67% (2/3)	69% (9/13)

AIH-related ACLF

A total of 10 (11.7%) patients presented with AIH-related ACLF (M:F = 4:6). Median age of presentation in males and females were 42 (IQR= 35-54) years, and 46 (31-59) years, respectively (p = 0.38) (Table 7). All patients received steroid therapy. Six patients (60%) went into remission within two weeks to five months and four others died during hospitalization. Of the patients who went into remission, four (75%) relapsed within three to six months of stopping the drugs. The sample size was too small to run the test for statistical significance for patients presenting with AHI-related ACLF and AS-AIH.

**Table 7:** Outcome of patients with autoimmune hepatitis-related acute-on-chronic liver failure ALP: alkaline phosphatase; ALT: alanine aminotransferase; AST: aspartate aminotransferase; IgG: immunoglobin G; INR: international normalized ratio

Patient characteristics	Male (n=4)	Female (n=6)
Age	42 (35-54)	46 (31-59)
Hemoglobin (g/dL)	9 (7.7-12.6)	12 (9.2-12.2)
Total leukocyte count (per/mm^3^)	6850 (3800-11500)	5250 (2881-8000)
Platelet count (lacs/mm^3^)	1.31 (1.05-1.61)	1.37 (1.25-1.58)
Total Bilirubin mg/ dL	18 (6.5-26)	20 (1.4-23)
Conjugated Bilirubin	12 (3.9-18)	15.2 (6.1-18.2)
AST (U/L)	182 (157-389)	89 (52-288)
ALT (U/L)	134 (102-223)	115 (32-209)
ALP (U/L)	112 ( 156-273)	137 (102-312)
Albumin (g/dL)	2.7 (2.5-3.65)	2.5 ( 2.3-4.0)
Protein	7.8 (7.2-7.77)	7.4 (5.8-8.0)
IgG	2640 (2177-3260)	2680 (2020-2910)
INR	1.7 (1.3-3.9)	1.82 (1.12-2.16)
Outcome	Male (n=4)	Female (n=4)
Remission	25% (1/4)	50% (3/6)
Mortality	50% (2)	33% (2/6)
Relapse	100% (2/2)	50% (2/4)

## Discussion

The overall incidence of AIH is increasing worldwide [10]. Despite a strong predilection for females, recent studies have shown increasing incidence among males in Asian countries [1]. Reports from India and similar other Asian countries support increasing male preponderance compared to Western countries (>90% females in Japan [6], 60-70% females in India [11]). This study also showed similar gender predilection (76% females) among patients with AIH. The median age of patients with AIH in India ranges from 37.90 ± 15.15 to 45.45 ± 16.15 years [11,12]. In our study, male patients presented earlier than females (not statistically significant), as found in a similar previous study by Chalabi et al. [3] (mean age, male vs female, 39 vs 49 years, respectively). The inclusion of an adult-only population can explain the higher mean age of male patients in this study. 

Liver-related decompensation was less common in patients with AIH and there was no statistically significant gender difference. It correlates with previous studies in which ascites, HE, and GI bleeding were present in 10-12% of patients [11]. One male patient (4%) and five (8.3%) female patients presented with ALF in this study which was similar to findings of previous studies where ALF or acute presentation as the first event is seen in around 3-6% and <10% of patients respectively with AIH [13-15,10]. A similar proportion of patients in both genders presented with ACLF (16%) and AS-AIH (24%) and this finding was also substantiated by previous studies [3]. Extrahepatic autoimmune diseases are prevalent in around 14-44% of AIH patients [5,16]. The most common autoimmune comorbidity in our study was autoimmune thyroiditis. This study showed a lower preponderance of extrahepatic autoimmune diseases among males (four patients in total, hypothyroidism in two, rheumatoid arthritis in one, and celiac disease in one) compared to females as found in similar previous reports [16]. Seronegative AIH was found exclusively in females (six patients (10%)), which corresponds to a previous report by Wang et al., in which, out of 167 patients, 17 (10%) were seronegative AIH and 16 (94%) of them were females [17]. The current study found cirrhosis in 27% of AIH patients with similar findings described in other studies [11]. Only a subset of patients respond to steroid and immunosuppressive therapy. Biochemical response was seen in 63% (complete response in 43%) of patients in this study as substantiated by previous similar Indian studies where steroid-induced remission was noted in 62-70% of patients [2,11,12]. Prior studies did not find any demographic, serological, or histological features predicting the relapse of AIH [18]. The present study found a higher relapse rate amongst male AIH patients; however, current data from India on this issue is limited. A previous study by Chalabi et al. showed that relapse (at least single) is more common in males as compared to females (71% vs. 55%) but despite that, they had better survival than females [3]. Amongst other factors, patients on steroids alone had a higher rate of relapse compared to those on steroids with immunosuppressives [19]. In contrast to that study, we had similar mortality among male and female patients with AIH.

The mechanism of higher relapses among male patients with AIH is not completely known. However, the presence of specific HLA has been often linked with relapses. In the study by Chalabi et al, 50% of male patients had extended human leukocyte antigen (HLA) haplotype A1-B8-DR3 [4]. Only one-fourth of females had the same haplotype in that study, which explains higher relapses among males as compared to females.

Though data on long-term mortality risk are limited from India, there is a twofold increased risk of death amongst AIH patients compared to the general population with a 10-year cumulative incidence of death of around 32%, as shown in a population-based cohort study from Sweden [20]. The current study cohort showed a five-year mortality of around 14% without gender predilection (Figure 2). Serum immunoglobulin-4 is associated with higher mortality in patients with autoimmune liver disease, especially in primary sclerosing cholangitis [21,22]. However, its significance in AIH remains to be explored. Hepatocellular carcinoma (HCC) is a rare complication of AIH as found in a recent study [23,24]. No patient developed HCC in our study. Patients who presented with AIH-ACLF and AS-AIH had a higher mortality corresponding to previous reports [25,26]. 

This study has a few limitations including being a single-center retrospective study with the inclusion of an adult-only population so it lacks generalizability. However, it is still among the few studies exploring predictors of relapse in AIH patients.

## Conclusions

Relapse in AIH is more common in male patients compared to female counterparts. Strict drug adherence and counseling are necessary to prevent relapse in patients with AIH. A similar proportion of male and female patients with AIH presented with cirrhosis, acute severe AIH, or AIH-related ACLF. Their biochemical, serological, and histological parameters are comparable; however, extra-hepatic autoimmune diseases were less common in male patients. The biochemical and clinical response at six months were seen in over 60% of patients in both groups. Overall five-year mortality was 14.1%, and no patient developed hepatocellular carcinoma. A prospective study with large data on this issue is warranted.
